# Effects of Aversive Stimuli on Prospective Memory. An Event-Related fMRI Study

**DOI:** 10.1371/journal.pone.0026290

**Published:** 2011-10-17

**Authors:** Massimiliano Rea, Stephanie Kullmann, Ralf Veit, Antonino Casile, Christoph Braun, Marta Olivetti Belardinelli, Niels Birbaumer, Andrea Caria

**Affiliations:** 1 Institute of Medical Psychology and Behavioral Neurobiology, University of Tübingen, Tübingen, Germany; 2 Department of Neurobiology, Italian Institute of Technology and Harvard Medical School, Boston, Massachusetts, United States of America; 3 Center for Neuroscience and Cognitive , Istituto Italiano di Tecnologia, University of Trento, Trento, Italy Systems@UniTn; 4 Center for Mind/Brain Sciences (CiMEC), University of Trento, Trento, Italy; 5 Department of Psychology 1, “Sapienza” University of Rome, Rome, Italy; 6 Istituto di Ricovero e Cura a Carattere Scientifico, Ospedale San Camillo, Venezia–Lido, Italy; University College London, United Kingdom

## Abstract

Prospective memory (PM) describes the ability to execute a previously planned action at the appropriate point in time. Although behavioral studies clearly showed that prospective memory performance is affected by the emotional significance attributed to the intended action, no study so far investigated the brain mechanisms subserving the modulatory effect of emotional salience on PM performance. The general aim of the present study was to explore brain regions involved in prospective memory processes when PM cues are associated with emotional stimuli. In particular, based on the hypothesised critical role of the prefrontal cortex in prospective memory in the presence of emotionally salient stimuli, we expected a stronger involvement of aPFC when the retrieval and execution of the intended action is cued by an aversive stimulus. To this aim BOLD responses of PM trials cued by aversive facial expressions were compared to PM trials cued by neutral facial expressions. Whole brain analysis showed that PM task cued by aversive stimuli is differentially associated with activity in the right lateral prefrontal area (BA 10) and in the left caudate nucleus. Moreover a temporal shift between the response of the caudate nucleus that preceded that of aPFC was observed. These findings suggest that the caudate nucleus might provide an early analysis of the affective properties of the stimuli, whereas the anterior lateral prefrontal cortex (BA10) would be involved in a slower and more deliberative analysis to guide goal-directed behaviour.

## Introduction

Prospective memory (PM) is the ability to remember executing an intended action at some future point in time [1], [2]. Experimental paradigms of prospective memory typically involve engaging participants in a primary task - ongoing task [3]-while at the same time asking them to perform an action upon perceiving a particular target item [2], [4]. Retrieving and executing the previously planned and encoded action, unrelated to the ongoing task, relies on prospective memory.

Behavioral studies revealed a prospective interference effect related to several ongoing activities and prospective cues that can be modulated by the task components features [4], [5], [6], [7], [8]. Several evidences, though not univocally, indicate that prospective memory is susceptible to emotional influences [9]. Thus far, most of the studies showed that enduring negative emotional states, fluctuations in mood, or clinically relevant affective disorders interfere with the ability to execute intended actions [10], [11], [12], [13], [14]. Only few studies investigated how prospective memory performance is modulated by the emotional valence of the intended action [15], [16], [17], [18], [19]. Meacham and Kushner [18] suggested that the probability to execute an intended discomfortable action diminishes with respect to a more neutral situation. However, aversive intentions seem to enhance memory retrieval but to delay execution. In accordance with this hypothesis, Clark-Foos and colleagues [17] showed that negative prospective cues compared to positive and neutral cues decrease PM performance. Similarly, Rendell and colleagues [19] investigating how emotional valence of the stimuli and age influence PM performance showed that young and old adults had better performance on positive than on both negative and neutral PM tasks. Although older compared to younger adults showed generally poorer levels of PM performance they demonstrated greater beneficial effects of positive valence. Furthermore, a recent study by Altgassen and colleagues [15] investigating the impact of emotional valence on event-based prospective memory performance in depression showed that healthy participants better remember positively valenced cues whereas this effect was absent in participants with depression. Both groups tended to be less accurate in response to negative PM cues with respect to positive and neutral cues and no significant difference has been found between them. On the contrary, a study from the same group of researchers [16] showed that, generally, emotionally valenced cues (positive and negative) increase prospective memory performance of younger and older adults. A decrease in performance associated with age was only observed when neutral (but not positive or negative) prospective cues were presented.

It has been proposed that modulatory effect of the emotional significance on prospective memory performance might be supported by the activity of the prefrontal cortex [17]. Previous neuroimaging and lesion studies indicated a key role of the prefrontal lobes, in particular the anterior prefrontal cortex (aPFC also referred as BA10), in prospective memory [3], [20], [21], [22], [23], [24], [25], [26], [27]. Burgess and colleagues [21] observed an increase of the BOLD response in the aPFC bilaterally under conditions in which a PM cue is expected, regardless of whether a PM cue is actually encountered. This response is specific to PM and is not related to working memory (WM). Reynolds and colleagues [28] demonstrated a dissociation between the sustained responses of the PM task performance, associated with enhanced activity in a network including the anterior prefrontal cortex, and the sustained responses associated with active maintenance in WM involving activity of dorsolateral PFC.

Previous studies investigated brain mechanism underlying PM using stimuli such as geometrical shapes [27] or emotionally neutral words [28]. Although behavioral studies clearly showed that prospective memory performance is affected by the emotional significance attributed to the intended action [17], [18], no study so far investigated the brain mechanisms subserving the modulatory effect of emotional salience on PM performance. The general aim of the present study was to explore brain regions involved in prospective memory processes when PM cues are associated with aversive stimuli. In particular, based on the hypothesised critical role of prefrontal cortex in prospective memory in the presence of emotionally salient stimuli [17], [29], [30] we expected a stronger involvement of aPFC when the retrieval and execution of the intended action is cued by an aversive stimulus. In fact, the emotional valence of stimuli seems to be processed by prefrontal cortex regions, whereas emotional arousal is mainly associated with activity in the amygdala [29]. Aversive stimuli compared to neutral stimuli require processing and response resources to be more intensely and urgently mobilized [31], [32]. In the presence of aversive stimuli a rapid subcortical mechanism preceding memory and decision processes would be subserved by the aPFC [33], [34]. LeDoux [33], [34] proposed that the subcortical pathway provides a quick analysis of the affective properties of stimuli that serves as an initial template for subsequent processing. Neuroimaging studies specifically reported frontal activity together with subcortical activations during processing of threat-related facial expressions [35], [36], [37]. Mühlberger and colleagues [37], investigating the neural effects of dynamic emotional facial expressions, reported that angry offsets were associated with stronger ventral striatum activation compared to angry onsets, in particular in the nucleus accumbens and the caudate nucleus bilaterally. Furthermore onsets of angry faces activated the lateral orbitofrontal cortex bilaterally, the left amygdala and the left insula.

Based on these results, we further hypothesised that in the presence of aversive PM cues subcortical mechanisms might precede memory and executive processes. An event-related fMRI design was adopted to test our hypotheses. Specifically, brain activity associated with prospective memory cued by aversive stimuli-angry faces – was compared to that associated with prospective memory cued by emotionally neutral stimuli. Pictures of facial expressions were used to trigger PM as human faces represent a unique category of biologically and socially important stimuli [38].

## Materials and Methods

### Ethics Statement

The study was approved by the ethics committee of the Faculty of Medicine of the University of Tübingen (Ethik-Kommission der Medizinischen Fakultät und am Universitätsklinikum Tübingen). All participants gave written informed consent to participate.

### Participants

Thirteen right-handed healthy participants (6 female; age range 23–35 years) with no history of neurological or psychiatric disorders gave their written informed consent to participate in this study. All subjects had normal or corrected-to-normal vision.

### Experimental protocol

**Figure 1:**
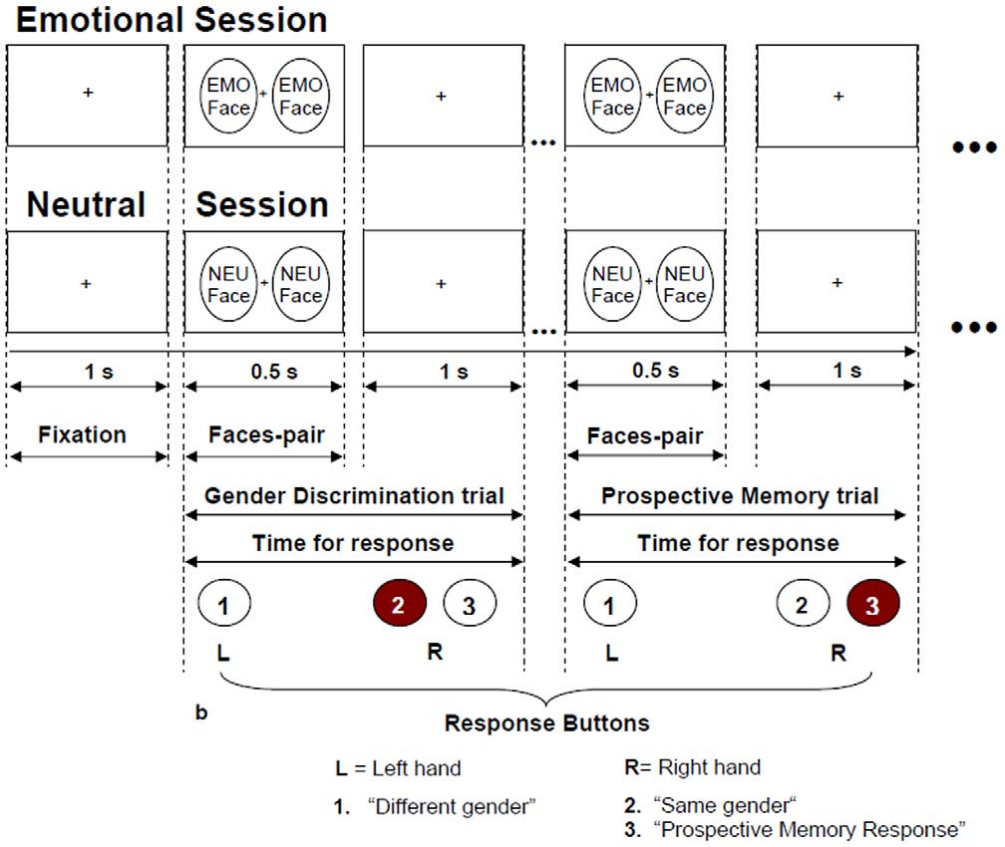
Task design. The experiment consisted of two sessions. A session presenting pairs of neutral facial expression (neutral) alternated to a session (emotional) presenting pairs of emotional facial expressions (angry, happy, surprise, and neutral). Participants were required to perform two tasks during both sessions. First, a gender discrimination task (ongoing task) during which they had to detect whether the gender of the two faces was the same or different. The button 2 was pressed in case of “same gender” and button 1 in case of “different gender”. Second, a PM task that consisted in detecting a specific face-pair target presented to the subjects prior to the session. When this combination occurred subjects were instructed to interrupt the gender discrimination task and press a different button (button 3, right hand) to indicate the detection of the target. During each trial (1.5 s) participants were shown a face-pair (0.5 s) followed by a fixation cross (1 s).

The experiment consisted of two sessions. A session presenting pairs of neutral facial expression - neutral (NEU) session - was alternated to a session where pairs of emotional facial expressions were presented - emotional (EMO) session. During the emotional session faces depicting angry, happy, surprise and neutral expressions were presented in a randomized order. The order of emotional and neutral sessions was counterbalanced across subjects. During each trial (1.5s) participants had to execute a button press response to the presentation of a face-pair (0.5s) followed by a fixation cross (1s) (Fig. 1). Participants were required to perform two tasks: an ongoing task and a PM task. The ongoing task was a gender discrimination task where subjects had to press a button with the right index finger in case of “same gender” (button 2) and a different button with left index finger in case of “different gender” (button 1). The PM task consisted in detecting a pre-specified face-pair target. When this combination occurred subjects were instructed to interrupt the gender discrimination task and press a third button (button 3) with the right middle finger.

Each session consisted of 806 trials whose 26 were PM trials. The interval between PM trials ranged between 30s and 1 minute. This PM's time presentation enables responses to multiple PM trials while maintaining an appropriate event rate [39], [40]. Prior to each session subjects memorized the PM cues and practiced with the task. Different face-pair targets (PM trials) were used for the NEU and EMO sessions. A single pair of faces with neutral expressions was presented for every PM trial of the NEU session and a single pair of faces with angry expressions was presented for every PM trial of the EMO session. The individual faces within the PM trials were not used for GD trials. Thus the PM and ongoing GD trials were not matched for stimulus repetition effects. Participants, debriefed at the end of the experiment, reported that the presentation of the emotional and neutral PM stimuli was unpredictable. The whole experimental protocol lasted about 48 minutes including a break of 8 minutes between the sessions.

### Visual Stimuli

The pictures were taken from the NimStim data set of adult faces showing the same individuals with a range of facial expressions [41]. The pairs of faces were composed from a pool of pictures of four different facial expressions (14 females and 14 males). Emotional expressions represented were happy, angry, surprise and neutral. Stimuli consisted of 1560 face-pairs, randomly selected. The two different PM trials were constructed using two pairs of neutral and angry facial expressions (four different faces) for the NEU and EMO session respectively (Fig. 1). The specific neutral and angry expressions selected had a high and comparable recognition rates [41]. Faces used in the PM trials were not used in the gender discrimination (GD) trials. Pictures were masked (hair removal) to minimize residual variance effect related to secondary attributes.

### fMRI acquisition

Data were acquired using a 3 Tesla Siemens MRI system (Siemens TIM Trio, Erlangen, Germany). A T1-weighted anatomical MR images was acquired in the first session using a 1 mm isotropic MPRAGE sequence with the following parameters: TR (repetition time) = 2300 ms; TE (echo time) = 3.03 ms; TI (inversion time) = 1100 ms; flip angle = 8°; FOV (field of view)  = 256×256 mm; matrix size = 256×256; number of slices = 160; slice thickness = 1 mm, bandwidth = 130 Hz/Px. Functional MR images were acquired using a gradient-echo planar imaging (EPI) aligned in axial orientation: TR = 2000 ms; TE = 30 ms; flip angle  = 90°; FOV = 192 mm; matrix size = 64; interslice gap = 3.75 mm; number of slices = 30; voxel size = 3×3×3 mm.

### fMRI data analysis

Functional data were pre-processed and analyzed using Statistical Parametric Mapping software package (SPM5; Welcome Trust Centre for Neuroimaging, London, UK). EPI volumes of the two fMRI sessions were realigned, co-registered to the T1 anatomical image, normalized to the Montreal Neurological Institute (MNI) reference space and spatially smoothed (9-mm FWHM Gaussian kernel). The time series in each voxel were high-pass filtered at 1/128 Hz to remove low frequency drifts.

For each participant, an analytic design matrix was constructed modeling onsets and duration of each trial as epochs convolved with a canonical hemodynamic response function. At the first level, for each single subject GD and PM conditions were modeled as separate regressors and interrogated to derive contrast images for second-level group analysis. All regressors were then incorporated into a general linear model (GLM). Motion correction parameters were included in the analysis as a covariate of no interest to model residual effects due to head motion. Contrast images were obtained subtracting GD trials from PM trials in both the EMO and NEU session. Only correct trials were considered in both EMO and NEU sessions and only GD trials of angry face pairs were modeled for the EMO session. PM and GD conditions were then matched according to the number of trials (26), gender and emotion of the face-pairs (only angry faces for the EMO session and only neutral faces for the NEU session), and hand used to respond (see Fig. 1). To enable appropriate event-related response estimation, only GD trials interspaced with an interstimulus interval ranging between 28.5s and 45s and PM trials with an interstimulus interval ranging between 30s and 60s were considered [28]. A minimal distance of 7 trials (10.5s) between GD and PM trials was also taken into account to avoid overlap of the BOLD response. A second-level mixed-effects analysis was performed to allow inferences across participants that generalize to the population. A first paired samples t-test compared PM trials with GD trials in the NEU session. This comparison aimed to investigate brain activations related to the PM task where no arousal or valence of facial expressions of either PM or GD trials was modulated. Moreover, this specific comparison allowed us to observe whether the use of human faces in a PM design induced brain activations consistent with those reported in previous studies adopting more abstract stimuli. A second paired samples t-test compared PM trials with GD trials in the EMO session. A further paired samples t-test explored our main contrast of interest (PM-GD)_EMO_-(PM-GD)_NEU_ using the contrast images (PM-GD)_EMO_ and (PM-GD)_NEU_ generated at the first level. The adopted between sessions paired samples t-test enabled to cancel out potential confounds either within or between sessions. A within session repetition effect might occur because of the different number of repetition of PM and GD trials, as the former were repeated whereas the latter were not. Additionally, we expected a difference in response to angry with respect to neutral stimuli and a possible general arousal effect associated with the presentation of emotional stimuli in the EMO session only. All these effects were mostly balanced either within (emotional valence of the stimuli and arousal effect) or between sessions (repetition effect) and were cancelled out in the main contrast of interest. However, a confound related to stimulus repetition might still affect our main contrast of interest (PM-GD)_EMO_-(PM-GD)_NEU_ in case of non-equivalent repetition effect in the EMO and NEU condition. Evidence exists about a reduced BOLD response due to repetition effect of face identity in fusiform cortex and posterior superior temporal sulcus (STS), whereas repetition of emotional expression lead to decreased activity in a more anterior region of STS [42].

A further paired samples t-test comparing GD_EMO_ with GD_NEU_ contrast images from single subject analysis aimed to observe potential differences in the GD trials between sessions. A statistical threshold of *p*<0.001 cluster extent k≥10 [43] across the whole brain was adopted. Significant brain activations were anatomically labeled using Automated Anatomical Labeling [44].

### Time course of hemodynamic activation within regions of interest

Event-related time courses (ERT) of the BOLD responses were extracted using the NERT4SPM toolbox (Axel Lindner & Christoph Budziszewski, Hertie Institute for Brain Research, Tuebingen, Germany, http://www.hih-tuebingen.de/en/sensorimotor-lab/nod-lab/). Regions of interest selection was based on brain clusters emerging as result of the main contrast of interest (PM-GD)_EMO_-(PM-GD)_NEU_. Average time courses of the emotional PM trials were calculated across all voxels within a spherical (6 mm radius) regions of interest (ROI) in the anterior prefrontal cortex area (BA10) (x, y, z = 37, 59, 8) and in the left caudate nucleus (x, y, z  = −19, 13, 11). Signal intensity within each ROI was normalized to a percent signal change scale based on a 3s pre-stimulus baseline (averaged across all trials) to decrease sensitivity to outliers. A paired t-test was performed to compare the peaks of time course in the caudate and BA10 region of each participant.

### Behavioral data analysis

Mean reaction times (RTs) and mean levels of response accuracy of EMO and NEU session were analyzed. Only correct PM and GD trials were considered in the analysis of the reaction times. Response accuracy was calculated for PM trials and for carefully matched GD according to gender, emotion of the faces, hand used to respond. Separate two-ways repeated measure ANOVAs were performed on RTs and accuracy, using trial and session as within subjects factors.

## Results

### Behavioral data

**Figure 2:**
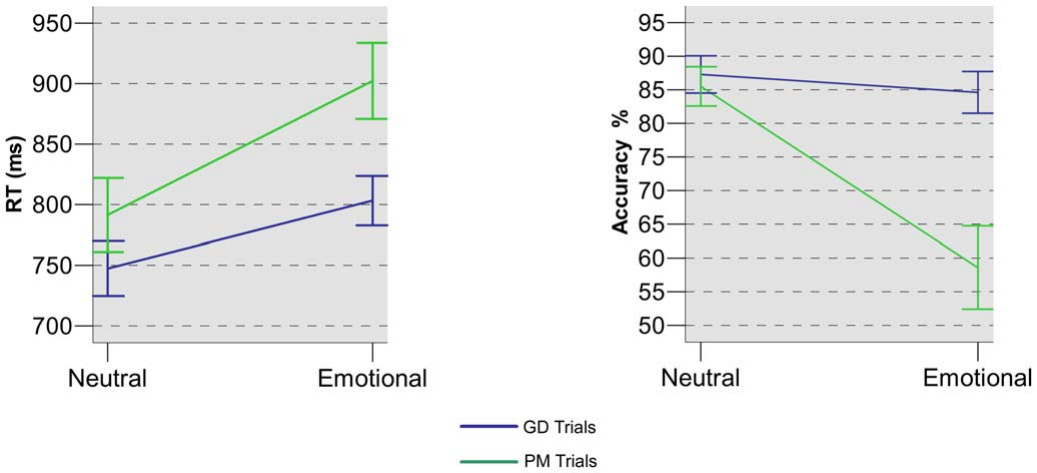
RT and accuracy for each condition. The graph on the left shows the mean reaction times (ms) (±1 SEM) of the PM trials and of GD trials in the emotional and neutral sessions. The graph on the right compares mean response accuracy (±1 SEM) between GD and PM trials of both sessions.

Mean levels of response accuracy and response time of the PM and control trials in both sessions are presented in Figure 2.

#### Reaction Times

Analysis of the mean reaction times revealed a significant main effect of the trial type (F_(1,12)_ = 13.363, *p*<0.005). Subjects were significantly slower in responding to PM trials than to GD trials. There was a significant main effect of session type (F_(1,12)_ = 18.557, *p*<0.005). The reaction times were significantly longer during the emotional session with respect to the neutral session. Notably, there was a significant interaction between trial type and session type F_(1,12)_ = 13.912, *p*<0.005). Paired-samples *t*-test comparisons revealed slower emotional PM trials than GD and neutral PM trials. Specifically, response time of emotional PM trials was slower than that of neutral PM trials (t_(12)_ = 5.55, p<0.001), emotional GD trials (t_(12)_ = 4.11, p  = 0.001) and neutral GD trials (t_(12)_ = 6.43, p<0.001).

#### Accuracy

Analysis of the accuracy revealed a significant main effect of the trial (F_(1,12)_ = 19.121, *p*<0.005). The mean performance of GD trials was significantly better than of PM trials. There was a significant main effect of the session F_(1,12)_ = 18.354, *p*<0.005). The mean performance was better during the neutral session with respect to the emotional session. There was a significant interaction effect between trials and session F_(1,12)_ = 13.301, *p*<0.005). Paired-samples *t*-test comparisons showed that participants made significantly more errors in the emotional PM trials compared to GD and neutral PM and trials. Specifically, the mean accuracy of emotional PM trials was lower than that of neutral PM trials (t_(12)_ = 4.46, p = 0.001), emotional GD trials (t_(12)_ = 4.7, p = 0.001) and neutral GD trials (t_(12)_ = 5.25, p<0.001).

### fMRI data

**Figure 3:**
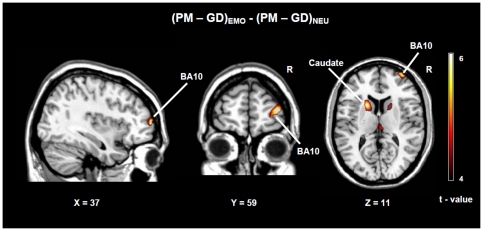
The main contrast of interest (PM-GD)_EMO_-(PM-GD)_NEU_. Statistical maps are superimposed on a standard single subject T1-weighted anatomical image. The colored bar represents the t values (p<0.001, cluster extent k≥10). Conditions label: PM-NEU = PM trials of the neutral session. GD-NEU: Gender discrimination trials of the neutral session. PM-EMO: Pm trials of the emotional session. GD-EMO: Gender discrimination trials of the emotional session.

The paired samples t-test comparing PM to GD trials in the NEU session revealed several brain activations including the left and right supramarginal gyrus (BA 40), the left and right inferior frontal gyrus (BA 47,48), the posterior cingulum and the anterior prefrontal regions (BA 9,10,46) (see Table 1 for the complete list). The paired samples t-test comparing PM to GD trials in the EMO session revealed, among others, brain activations in the middle temporal gyrus (BA 21), inferior parietal gyrus (BA 40), thalamus and caudate nucleus, middle cingulum (BA 32) and the anterior prefrontal regions (BA 9,10,46) (see Table 2 for the complete list). The results of PM vs GD trials contrasts of both EMO and NEU sessions are in line with findings from previous PM studies [27], [28], [45]. Furthermore, the main contrast of interest (PM-GD)_EMO_-(PM-GD)_NEU_ revealed two clusters of brain activity in the right lateral prefrontal cortex (BA 10) and in the left caudate (MNI peak maxima BA10 x, y, z = 37, 59, 8, z = 4.35; left caudate x, y, z  = −19, 13, 11, z = 4.12; see Fig. 3 and Table 3). The paired-samples t-test comparing GD_EMO_-GD_NEU_ and GD_NEU_-GD_EMO_ did not show any activation using a threshold significance p <0.001 cluster extent k≥10.

**Table 1:** Significant brain activations of the contrast (PM-GD) within the NEU session.

			MNI coordinates			
Brain Regions	H*	Voxels	X	Y	Z	Z value
**(PM-GD)_NEU_**						
Inferior parietal gyrus (BA 2)	L	2612	−56	−30	52	5.67
Inferior parietal gyrus (BA 1)	L		−56	−26	38	5.57
Postcentral gyrus (BA 3)	L		−36	−33	49	5.51
Inferior parietal gyrus (BA 40)	R	1146	46	−36	−49	5.07
Supramarginal (BA 40)	R		63	−23	41	4.98
Inferior parietal gyrus (BA 40)	R		36	−43	49	4.73
Thalamus	L	216	−6	−26	−11	4.61
Hyppocampus	R		30	−23	−8	4.36
Lingual gyrus	R		16	−33	−8	4.80
Inferior frontal operculum (BA 48)	L	335	−43	10	15	4.43
Precentral gyrus (BA 6)	L		−56	3	38	4.34
Insula (BA 48)	L		−36	13	−4	4.11
Calcarine sulcus	R	218	26	−73	−11	4.38
Cerebellum	R		20	−56	−19	4.35
Cerebellum	R		30	−30	−30	3.78
Inferior frontal operculum (BA 47)	R	426	49	16	0	4.30
Inferior frontal pars triangularis (BA 47)	R		46	26	0	4.02
Posterior cingulum (BA 23)	R	320	3	−36	26	4.22
Posterior cingulum (BA 23)	L		−7	−43	22	3.98
Middle cingulum	L		−10	−30	45	3.92
Middle frontal gyrus (BA 9)	L	92	−33	33	30	4.15
Middle frontal gyrus (BA 9)	L		−40	26	45	4.11
Middle frontal gyrus (BA 46)	R	77	33	36	34	4.00
Middle frontal gyrus (BA 9)	R		33	49	38	3.19
Middle frontal gyrus (BA 10)	R		28	56	30	3.14

*H = Hemisphere.

P<0.001, cluster extent k≥10. Coordinates are in MNI stereotaxic space [70] and labelled anatomically according to Automated Anatomical Labeling [44].

**Table 2:** Significant brain activations of the contrast (PM-GD) within the EMO session.

			MNI coordinates			
Brain Regions	H*	Voxels	X	Y	Z	Z value
**(PM-GD)_EMO_**						
Middle frontal gyrus (BA 10)	R	26	26	59	4	5.43
Inferior orbitofrontal gyrus (BA 47)	R	321	46	23	−4	5.42
Inferior orbitofrontal gyrus (BA 47)	R		36	23	−8	5.31
Insula (BA 47)	R		33	16	−19	5.17
Inferior parietal gyrus (BA 40)	L	597	−49	−40	56	5.39
Inferior orbitofrontal gyrus (BA 2)	L		−46	−40	45	5.11
Superior parietal gyrus (BA 7)	L		−33	−53	71	5.10
Middle cingulum	R	104	0	−26	30	5.06
Middle cingulum	R		7	−43	38	3.81
Superior temporal pole (BA 38)	L	667	−33	13	−26	4.99
Thalamus	L		−7	−7	4	4.92
Caudate nucleus	R		16	7	11	4.85
Precuneus (BA 7)	L	209	−3	−73	38	4.82
Precuneus (BA 7)	L		−10	−66	30	4.50
Precuneus (BA 7)	R		13	−66	30	4.49
Angular gyrus (BA 39)	R	286	40	−66	45	4.75
Supramarginal gyrus (BA 40)	R		63	−46	30	4.62
Supramarginal gyrus (BA 40)	R		66	−36	38	4.51
Precentral gyrus (BA 9)	L	27	−59	7	38	4.69
Middle frontal gyrus (BA 8)	R	39	40	23	45	4.45
Middle frontal gyrus (BA 9)	R		43	33	34	4.38
Cerebellum	R	21	7	−53	−22	4.40
Cerebellum	R	18	23	−53	−22	4.39
Supplementar motor area (BA 6)	R	130	3	13	49	4.36
Middle cingulum (BA 32)	R		10	33	30	4.33
Middle cingulum (BA 32)	R		10	16	41	4.29
Middle frontal gyrus (BA 46)	L	34	−40	30	41	4.36
Middle temporal gyrus (BA 21)	R	40	66	−53	4	4.18
Middle temporal gyrus (BA 21)	R		69	−46	−4	4.13
Middle temporal gyrus (BA 21)	R		63	−36	−4	3.94
Frontal superior medial (BA 10)	R	42	10	49	4	4.11
Anterior cingulum (BA 24)	L		−3	36	4	4.05
Anterior cingulum (BA 32)	R		3	43	8	3.95

Notes: see Table 1 for specification and abbreviations.

**Table 3:** Significant brain activations of the contrast (PM-GD)_EMO_-(PM-GD)_NEU_.

			MNI coordinates			
Brain Regions	H*	Voxels	X	Y	Z	Z value
**(PM-GD)_EMO_-(PM-GD)_NEU_**						
Middle frontal gyrus (BA 10)	R	37	37	59	8	4.35
Caudate nucleus	L	99	−19	13	11	4.12
Putamen	L		−23	18	0	3.76
Thalamus	L		−4	−4	4	3.60

Notes: see Table 1 for specification and abbreviations.

### Time course of the hemodynamic responses

**Figure 4:**
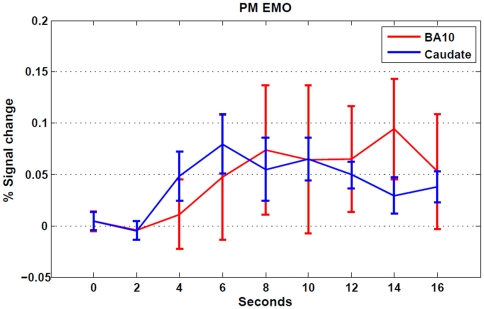
Time course of the hemodynamic responses derived from area BA10 and caudate nucleus. Time courses of BOLD signal change (±1 SEM) during PM-EMO trials were extracted from area BA 10 (x = 37, y = 59, z = 8) and from left caudate nucleus (x = −19, y = 13, z = 11). The figure shows an earlier activation of the caudate nucleus (blue line) followed by the activation of area BA10 (red line) during PM trials of the EMO session.

The time trajectories averaged across all subjects of the normalized time courses were derived from the anterior right prefrontal cortex (BA10) and left caudate nucleus during the emotional PM trials (Fig. 4). Visual inspection revealed a different peak latency of the two trajectories derived from the PM-EMO trials. A paired-samples t-test revealed a significant earlier peak of activation in the caudate nucleus (peak time bin: 6.53 s) compared to BA10 (peak time bin: 8.78 s) region (t_(12)_ = 3.47 p = 0.005).

## Discussion

The general aim of the present study was to investigate brain regions involved in prospective memory processes when aversive stimuli cue the retrieval and execution of a previously planned action, with a specific focus on the role of aPFC. Based on previous studies we hypothesized that aversive stimuli would have decreased PM performance. Moreover we expected a greater involvement of aPFC activity due to the association of PM with emotionally salient stimuli. To this aim BOLD responses of PM trials cued by aversive facial expressions were compared to PM trials cued by neutral facial expressions. A novel finding of this study is that PM task prompted by aversive stimuli is specifically associated with activity in the right lateral prefrontal area (BA 10) and in the left caudate nucleus.

At a behavioral level, participants' responses (RTs) during emotional PM trials were slower and less accurate with respect to neutral PM and GD trials. These results show that the negative valence of PM cues negatively affect prospective memory performance in comparison with neutral PM cues. The few studies that investigated the effect of emotional valence on PM performance partially converge on showing a facilitatory effect of positive valence associated with prospective cues as opposed to negative and neutral valence [15], [16], [17], [19]. Moreover, it has been posited that aversive stimuli by implying an increased attentional load would impair successful prospective memory performance [17]. Several studies investigated whether attention is selectively modulated by different facial expressions (for a review see [46]). Öhman and colleagues [47] showed that participants had more difficulty in disengaging attention from angry faces compared to neutral or happy faces. In line with this finding van Honk et al. [48], using an emotional Stroop task, found that naming the colour of an angry face took longer than naming the colour of a neutral face. Moreover, Fox et al. [49] suggested that the presence of an angry face had a strong impact on the disengagement of attention by means of spatial cueing paradigm. In agreement with these findings and with a recent observation of a reduced accuracy in response to negative PM cues compared to positive and neutral PM cues [15], we observed that aversive stimuli (angry faces) interfere with successful prospective memory performance.

In the present study, to increase ecological validity [38] prospective memory was studied adopting pictures of human faces. Functional data of the NEU session confirmed the validity of our approach. In fact, a brain network that included the supramarginal (BA 40) and the inferior frontal gyri (BA 47,48) bilaterally, the posterior cingulum and the anterior prefrontal regions (BA 9,10,46) was previously reported in neuroimaging studies on PM [21], [27], [28].

Whole brain analysis directly comparing the emotional to the neutral session showed that PM task cued by aversive stimuli involves the right lateral prefrontal area (BA 10) and the left caudate nucleus. The observed increased activation of area BA 10 during prospective memory in the presence of aversive stimuli suggests a higher recruitment of attentional resources. Brodmann area 10, has been associated with high level cognitive processes like internal thought [50], changing between externally driven and internal mental processes [45], integration of several mental processes [26], [51] or hierarchical processing of several tasks [52], [53]. Lateral and medial prefrontal regions have been implicated in cognitive control relevant to emotion, such as suppressing the processing of emotional information or controlling emotional feelings [54], [55]. An fMRI study [56] showed that lateral prefrontal cortex is involved in establishing increased attention control over expected threat-related distractors such as angry faces. Higher activity in response to negative stimuli compared to positive stimuli has been found in sub-regions of the right ventrolateral PFC [30]. It has been demonstrated that prefrontal cortex is also recruited during memory encoding [57], [58], [59]. Meta-analysis showed that anterior prefrontal cortex is consistently active during episodic retrieval [50]. Several studies confirmed the involvement of the prefrontal cortices in cognitive control over memory processes that can influence, and in turn are influenced by, emotional processes (for a review see [54]). Furthermore, inhibition of emotional memory retrieval involves right lateral PFC mechanisms [60]. These convergent evidences indicate that anterior lateral prefrontal cortex (BA10) might be critical when prospective memory occurs in the presence of emotional stimuli. Two different processes might underlie the larger involvement of the aPFC: a compensatory and/or an intrusive mechanism. A compensatory mechanism would occur to overcome the interference effect due to the recruitment of additional brain regions associated with the processing of emotionally salient cues. In addition, an intrusive mechanism might result from the simultaneous recruitment of the prefrontal regions to subserve PM performance and processing of aversive stimuli.

Our results also highlighted an activation of the left caudate nucleus. This region might support a fast analysis of the affective properties of the aversive stimuli that serves as an initial template for subsequent processing and memory response [37], [61]. Alternatively, caudate nucleus might subserve response strategies (avoidance response) upon an aversive stimulus [62], [63] as in the case of PM cued by angry faces.

In a recent fMRI study [61] a old/new memory task was used to assess differences in brain responses to recently acquired familiarity of face identities relative to unfamiliar faces. Authors showed that the activity in the left caudate nucleus was involved in familiar face recognition and specifically modulated by the aversive valence of the stimuli (angry face) previously encountered. Another study [37] investigated the neural effects of the dynamic onset and offset of emotional facial expressions. Authors showed that angry offsets were correlated with stronger ventral striatum activation, in particular in the nucleus accumbens and caudate nucleus bilaterally, compared to angry onsets. Further evidences demonstrated that the caudate nucleus shows greater activation in response to negative pictures with respect to neutral pictures [62], [63]. Moreover, the dorsal striatum (caudate nucleus and putamen) activates defense/withdrawal motor programs [64] and stimulates autonomic/motor actions to cope with unpleasant events [63], [65].

Finally, we observed a temporal shift between the responses of the aPFC and the caudate nucleus during PM trials cued by aversive stimuli. Specifically, the activation of the caudate nucleus preceded that of aPFC. These results might support our hypothesis that in the presence of aversive PM cues a rapid subcortical mechanism precedes memory retrieval and execution. This would have an obvious adaptive and evolutionary advantage: the consequences of a negative event are often much more dramatic than the consequences of ignoring or reacting slowly to neutral or even appetitive stimuli [31], [32]. Moreover, fast identification of aversive stimuli allows early activation of defense systems [34], [47]. Thus, the caudate nucleus would provide an early analysis of the affective properties of the stimuli that serves as an initial template for a subsequent processing, whereas the anterior lateral prefrontal cortex (BA10) would be involved in a slower and more deliberative analysis to guide goal-directed behaviour.

However, regional differences in the hemodynamic response [66], [67] might also underlie the observed time lag between prefrontal cortex and caudate nucleus, although intersubject variability is more commonly observed than brain regions variability [68]. Moreover, variability in the time-to-peak as well as shape, magnitude etc. of the hemodynamic response function across subjects and brain regions may arise from multiple factors such as neural activity differences, vasculature differences, global magnetic susceptibility etc. that are not easily dissociable [69]. One further issue is that the PM and ongoing GD trials were not matched for stimulus repetition effects. Although any common effect of stimulus repetition between the neutral and emotional conditions should be subtracted out in the interaction analysis, results might still reflect differential effects of stimulus repetition between the two conditions.
